# The Use of Three-Dimensional Images and Food Descriptions from a Smartphone Device Is Feasible and Accurate for Dietary Assessment

**DOI:** 10.3390/nu16060828

**Published:** 2024-03-14

**Authors:** Jeannette M. Schenk, Alanna Boynton, Pavel Kulik, Alexei Zyuzin, Marian L. Neuhouser, Alan R. Kristal

**Affiliations:** 1 Cancer Prevention Program, Division of Public Health Sciences, Fred Hutchinson Cancer Center, Seattle, WA 98109, USA; aboynton@fredhutch.org (A.B.); mneuhous@fredhutch.org (M.L.N.);; 2 Allen Institute, Seattle, WA 98109, USA; pavel.kulik@alleninstitute.org; 3 Illionix Product Development, Seattle, WA 98125, USA; azyuzin@illionix.com

**Keywords:** dietary assessment, dietary intake, food records, technology-assisted dietary assessment, public health, mHealth

## Abstract

Technology-assisted dietary assessment has the potential to improve the accuracy of self-reported dietary intake. This study evaluates MealScan3D (MS3D), a mobile device-based food recording system, which uses three-dimensional images to obtain food volumes and an application to capture algorithm-driven food intake data. Participants (*n* = 179) were randomly assigned and trained to record three meals using either MS3D or a written food record (WFR). Generous amounts of standardized meals were provided, and participants self-selected portions for each food. The weights of provided and uneaten/leftover foods were used to determine true intake. For total energy intake (three meals combined), validity (Pearson correlation) was significantly higher for MS3D vs. the WFR (*p* < 0.001); when interpreted as the percentage of variance in energy intake explained, MS3D explained 84.6% of true variance, a 25.3% absolute and 42.6% relative increase over the 59.3% explained by the WFR. For 9 of 15 individual foods, the Pearson correlations between true and reported portion size estimates were significantly larger for MS3D than the WFR. Bias was smaller (intercepts were closer to the means) for 9 of 15 foods and the regression coefficients for 10 of 15 foods were significantly closer to 1.0 in the MS3D arm. MS3D is feasible for dietary assessment and may provide improvements in accuracy compared to WFRs.

## 1. Introduction

Despite decades of methodological research, measuring nutrient intake among free-living individuals remains challenging. Methods based on recovery biomarkers, such as urinary excretion of sodium or doubly labeled water (for energy), are quite accurate, but they are only available for few nutrients and not useful in large studies due to their procedural complexity and cost [1]. In contrast, methods based on self-reports of food intake, such as 24 h recalls, food records, or food frequency questionnaires, are comparably simple to administer and inexpensive, but their validity for most nutrients is poor. For example, when using multiple-day diet records or 24 h recalls, correlations between true and self-reported energy intake are rarely higher than 0.4–0.6 and they are further affected by systematic measurement error [2,3]. Poor dietary measurement in observational research makes it difficult to detect associations between diet and disease risk [4], and in clinical applications, it limits the ability of nutritionists to both develop individualized dietary change interventions and evaluate intervention effectiveness.

Many scientific groups have developed systems that incorporate image-based technologies available on smartphones and other mobile digital devices to improve dietary self-report. In general, these have focused on the combination of (1) digital food images to capture the amounts of foods consumed and (2) real-time, technology-assisted methods to capture food descriptions. Previously described methods for measuring food portion size using digital food images, including those proposed, in development, or not widely adopted after evaluation, have been based on trained raters [5,6,7,8,9,10,11,12], stereo vision techniques using multiple images [13,14,15,16], depth and pixel count [17,18], structured light systems [19], and pre-defined 3D geometric models or shape template fitting [15,20,21,22,23]. Manual analysis involving a trained rater can produce reasonably accurate food volume estimates but is extremely burdensome [24,25,26,27]. Semi-automatic and automatic approaches for portion size estimation are promising, though approaches differ substantially, and technologic advances may be needed to improve their utility. Simple digital images without a fiducial marker are not very useful because variations in camera angle and distance from food preclude calculating food volume from an image alone. It also appears that more sophisticated cameras and image processing algorithms will be needed to solve problems such as poor lighting, non-standard plate and food sizes and shapes, and food additions such as sauces and condiments, before calculated food volume can replace self-reported portion size. Almost all image-based approaches have also incorporated some type of technology-assisted method to collect food descriptions, which have ranged from simple annotations of images [6,8,11,26,27] to automatic food identification [13,14,28,29,30]. The technology-aided collection of food descriptions could yield data superior to those from unstructured food descriptions written into a paper booklet, although few have examined this question.

MealScan3D (MS3D), developed by Illionix Product Development (Seattle, WA USA) in collaboration with the Nutritional Assessment Shared Resource (NASR) at the Fred Hutchinson Cancer Center (Fred Hutch), is a novel smartphone-based system for collecting food records which combines the use of an infrared structured light imaging system to capture three-dimensional images of foods and a systematic, algorithm-driven approach to capture detailed food descriptions. We report here the evaluation of MS3D using a randomized design to compare the quality of dietary data captured in MS3D with that from a written food record (WFR), using true (weighed) food intake as the criterion measure for validity. The primary hypothesis is that the validity of total energy intake measured by MS3D will be higher than the validity of energy measured from a WFR. Secondary analyses test whether food volume estimation using three-dimensional imaging is superior to self-report, and whether the algorithm-driven collection of food descriptions is superior to hand-written descriptions in a structured paper booklet.

## 2. Materials and Methods

### 2.1. Study Participants

**Figure 1:**
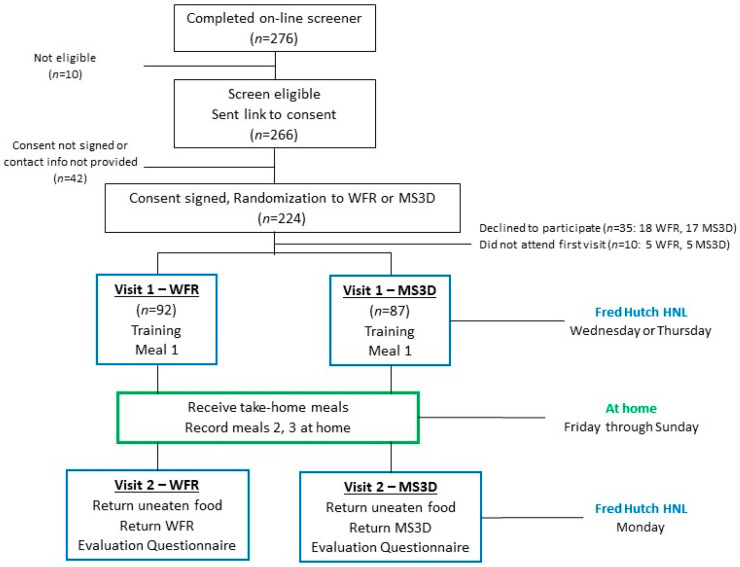
Study schedule and participant flow in the MealScanner Evaluation Study.

This convenience sample of male and female adult participants was recruited from employees of Fred Hutch and its partner institution, the Seattle Cancer Care Alliance, using notices posted on employee bulletin boards and an internal website. Interested individuals visited the study website to learn about the study details and complete a brief, web-based questionnaire to screen for eligibility. Eligibility criteria were ≥18 years of age; fluency in English; and willingness to eat the foods provided in the study, be randomly assigned to either arm, and complete all study activities. Those with food allergies, no access to a refrigerator and microwave, or with formal academic training in nutrition science were excluded. After eligible respondents completed an online consent form, they were randomly assigned to the MS3D or WRF arm, using permuted blocks of sizes 4 and 6, and scheduled for the first in-person study visit (Figure 1). Because the first visit included a group training session and lunch eaten in a common space, day-one visits were conducted by randomization arm; however, to minimize drop-out, participants were not informed of the arm into which they were randomized until the first visit. Informed consent was obtained from all participants, and the study protocol was approved by the Fred Hutch Institutional Review Board.

### 2.2. Participant Activities

At the first visit, participants completed a demographic questionnaire and attended a 30 min Registered Dietitian-led group training session on how to record meals using MS3D or a WFR. Both arms (MS3D and WFR) received comparable training on describing foods and estimating portion sizes and were provided the same reference guide (Serving Size booklet: https://www.fredhutch.org/content/dam/www/research/divisions/public-health-sciences/nutrition-assessment/serving%20size%20booklet_english_v5_web.pdf accessed on 21 February 2024) to assist with portion size estimation. Participants were asked to eat and record 3 study meals using their assigned mode (MS3D or WFR). The first meal was prepared by the Fred Hutch Human Nutrition Laboratory (HNL) and served onsite after training. Meals 2 and 3 were prepared and packaged by a local food delivery provider (Chicken Soup Brigade) for consumption at the participant’s home. Storage and preparation instructions were provided for home meals. On the Friday following visit one, participants received a cooler containing meals 2 and 3 (via delivery to home or workplace) to be eaten over the weekend. On Mondays, participants returned the cooler along with foods not consumed and the MS3D or WFR, and they completed a short questionnaire on their experience. Participants received USD 25 for completing the study.

All participants, regardless of their assigned study arm, were fed the same 3 study meals, designed to mimic ad libitum dietary intake. Each meal was standardized to include a meat, carbohydrate, vegetable, and dessert. The meal descriptions, along with the mean portions of foods provided and their total energy per 100 g, are given in Supplemental Table S1. The foods provided were considerably more than a single person would eat at a meal, which allowed the participants to choose varied portion sizes. For each meal, the participants were provided their own set of pre-weighed foods, each in its own individual serving dish (Meal 1) or container (Meals 2 and 3). Participants placed desired amounts of foods on their plate. After eating, any foods left were carefully separated and returned to the serving dishes/containers by either HNL staff (Meal 1) or participants (Meals 2 and 3) before uneaten foods were weighed back by HNL (Meal 1) or Chicken Soup Brigade staff (Meal 2). Participants were asked to record only the meals/foods provided by the study.

### 2.3. MealScan3D System

**Figure 2:**
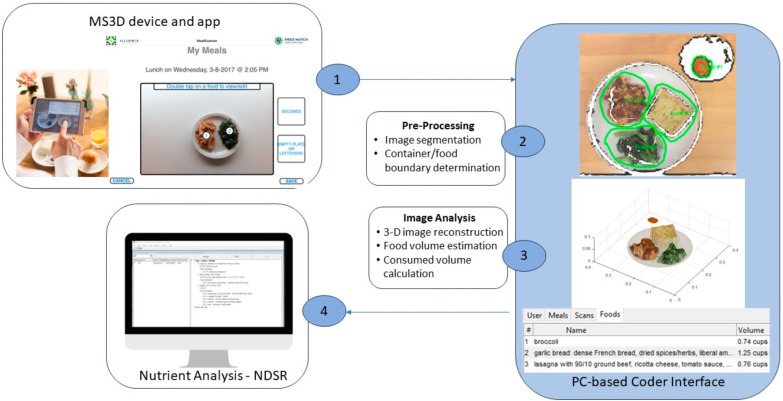
MealScan 3D Food Volume Estimation System. Step 1. Participant captures images and documents within the MS3D mobile app; Step 2. Images are processed to segment foods and identify container/food boundaries; Step 3. Images are analyzed to estimate food volumes; Step 4. Food descriptions and volumes entered into NDSR software.

MS3D is a food volume estimation system based on three-dimensional (3D) scanning and 3D model processing (Figure 2). The MS3D system consists of the hardware for data collection, an algorithm-driven user interface that manages data collection, and a coder interface that supports semi-automated image processing and data reformatting to generate a dataset suitable for dietary analysis. The MS3D hardware is an infrared camera (Occipital Structure Sensor manufactured by Occipital Inc.; Boulder, CO, USA) mounted to an iPad mini (Apple; Cuppertino, CA, USA) which is used to obtain 3D scans. The MS3D mobile software (Version 1) application, developed on the Apple iOS platform, consists of an algorithm-driven user interface to manage the collection of meal scans and corresponding food descriptions. A PC-based coder interface (CI) written in MATLAB (https://www.mathworks.com/products/matlab.html, Mathworks, Inc.; Natick, MA, USA) provides assisted 3D model processing to segment and calculate food volumes in each scan. (A detailed description of the MS3D methods for food volume estimation can be found in Appendix A).

At the beginning of each study meal, participants used the MS3D application to scan their plate containing the self-served portions of study foods. Images from this scan were recorded simultaneously in two- (2D) and two-dimensional (3D) formats. To record the initial food descriptions, users tapped each food in the 2D image, which opened a pop-up screen with prompts to enter the food name, a detailed description, the preparation method, condiments or other additions, and the estimated portion size in household measures. Options were available to capture this information via text or voice recording. Participants were provided with a serving size booklet that includes measuring tools and photographs to help estimate portion size (https://www.fredhutch.org/content/dam/www/research/divisions/public-health-sciences/nutrition-assessment/serving%20size%20booklet_english_v5_web.pdf, accessed on 21 February 2024). If a participant had second helpings, they captured an image of the plate containing the second helpings and the pop-up screen was used to report the estimated portion size(s). At the end of each study meal, participants captured an image of the plate containing any foods not eaten or the empty plate, and they again used the pop-up screens to report the portion sizes of foods not consumed. Upon completion of the study meals and the return of MS3D, data were transferred to a standard personal computer for processing. (Additional details about the MS3D hardware, image capture, and process to calculate food volumes are provided in Appendix A. Images of the MS3D hardware and screenshots of the application are available at http:www.mealscan3d.com, accessed on 21 February 2024).

### 2.4. Written Food Record

The written food record (WFR) booklet and training protocol were developed at Fred Hutch and designed to be used without interviewer documentation after completion [31]. The form is a 20-page booklet with an example of a correctly entered meal in the front (https://www.fredhutch.org/content/dam/www/research/divisions/public-health-sciences/nutrition-assessment/multiple%20day%20food%20record_undocumented_english_v4_web.pdf, accessed on 21 February 2024), which is distributed with the same serving size booklet. Participants were asked to record food descriptions, the preparation method, and the estimated amount of food consumed in household measures.

### 2.5. MealScan3D Data Processing

MS3D data were processed by Registered Dietitians with special training in both dietary assessment and in using the MS3D coder interface. They first examined the three-dimensional food images and, if necessary, adjusted the software-generated boundaries around each food manually. They then read or listened to the description of each meal and generated a list of foods consumed in a format suitable for entry into the Nutrient Data System for Research (NDSR), for example, food description, preparation, additions, and reported portion size. They then entered these data into NDSR [Version 2017], generating one nutrient output dataset using the volumes measured by the MS3D three-dimensional image. A separate dataset with the identical food descriptions was also generated using only self-reported portion sizes (ignoring the MS3D-measured volumes).

### 2.6. True Intake

For each individual food, the true amount consumed was estimated by subtracting the weight of the uneaten/returned foods (including plate waste) from the served food weight. All food items were weighed in their serving dishes/containers before and after consumption.

### 2.7. Energy

MS3D and WFR data were entered into NDSR directly following a standardized protocol. The nutrition coder (KJ) entering the data was blinded to the true food descriptions and was careful to use the food descriptions provided by study participants, even if inconsistent with the food images (MS3D only). A set of established coding rules was used to handle incomplete or missing information. True intake was entered as a separate record into NDSR by a different coder (AB), using the recipes for each prepared food.

### 2.8. Statistical Analysis

Descriptive analyses were used to examine differences in participant characteristics across treatment arms, using chi-square tests to determine statistical significance. Descriptive analyses also examined the mean differences between true and reported energy and food portion sizes. One-sample *t*-tests were used to determine whether the mean differences between true and reported total energy and food portion sizes within each arm differed significantly from zero. Two-sample *t*-tests were used to test whether the absolute values of these mean differences were significantly different across arms. Bland–Altman analyses were conducted to compare the limits of agreement between reported and true total energy between MS3D and the WFR, and to evaluate whether the bias in estimating total energy intake differed by the amount of energy intake [32].

The primary analyses tested whether the associations of reported total energy and food portion sizes with their true values differed between the MS3D and WFR study arms. Analyses of total energy included all participants. Analyses of individual foods examined portion sizes only, and these excluded observations with missing values. Pearson correlation coefficients (r) between reported and true values were used as a measure of validity and r^2^ as a measure of shared variance; tests for differences between correlation coefficients are based on Fisher’s Z transformation. We also give results from linear regression models predicting true intake (dependent variable) from reported intake (independent variable), where reported intakes of total energy and individual food volumes are centered on the mean. From these models, we interpret the intercepts as a measure of bias and regression coefficients as a measure of the slope between measured and true intake, for which perfect prediction would yield intercepts equivalent to the measured mean intake and regression coefficients of 1. An additional model combining data from both arms included an indicator for study arm (coded 0 or 1) and the interaction of reported intake and study arm. The coefficient for the interaction term tested for differences in slopes between the two arms.

Two additional secondary analyses were completed using the correlation and regression models as described above. The first examined whether energy intake and portion sizes were more accurately captured using the three-dimensional imaging compared to self-report, using data from the MS3D arm only. This analysis followed the statistical approach used for the primary analysis but was modified to account for the lack of independence between observations. Differences between correlations used tests of dependent correlations [33] and regression models used the seemingly unrelated regression (SUR) method within the SYSLIN procedure in SAS [34]. The other secondary analysis examined whether self-reported intake was better captured using the structured algorithms within the MS3D interface compared to the traditional WFR. For this analysis, we ignored the three-dimensional images in the MS3D arm and used only the self-reported portion sizes for analysis.

## 3. Results

The demographic characteristics of study participants are given in Table 1. The mean age was 33.6 years (range 19 to 74), 73% were female, and only 13% had no post-high school education. Most participants were white (65%) or Asian (22%), and approximately two-thirds were responsible for all or most of the food preparation and meal planning in their households. There were no differences in these characteristics between study arms, with the exception of sex; fewer participants in the MS3D arm compared to the WFR arm were male (21% vs. 34%).

Table 2 gives the means of true and reported intakes, the means of differences between true and reported intakes, and a test of whether the absolute values of these differences is different across arms. Individual differences between reported and true total energy intake in MS3D and WFR participants ranged from −688 to 1627 and from −2215 to 1089, respectively. Total energy intake was overestimated by a mean of 120 kcal in the MS3D arm and underestimated by a mean of 171 kcal in the WFR arm; the absolute value of mean differences between true and reported intakes was 51 kcal smaller in the MS3D arm compared to the WFR arm (*p* < 0.0001). For individual foods, the mean differences between true and reported portion sizes differed significantly between arms for 11 of 15 foods; for six foods (all vegetable and starch sides, garlic bread, and rice pudding), they were smaller in the MS3D arm, and for five foods (main entrees, butters, and one sauce), they were smaller in the WFR arm.

**Figure 3:**
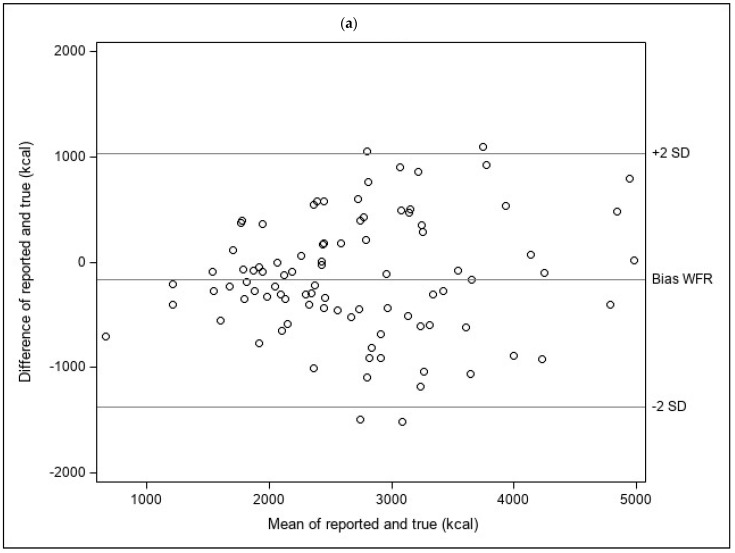

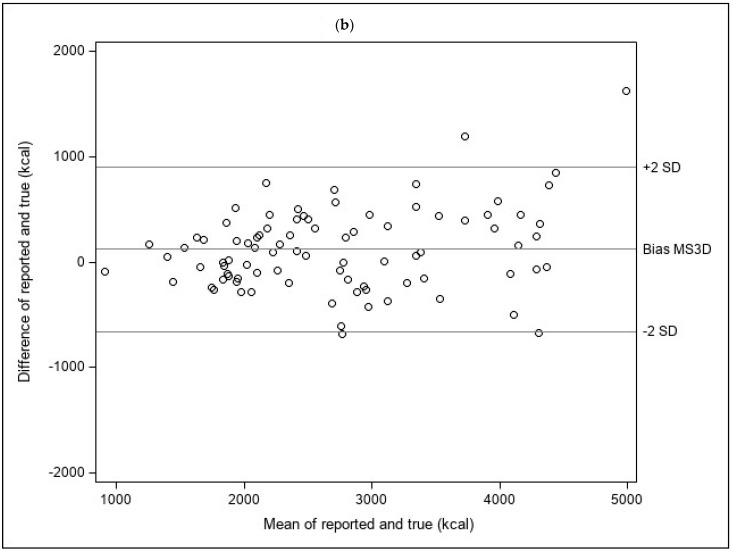
(**a**) Bland-Altman plots showing the difference in kcal between the reported and true intake for the written food record. (**b**) Bland-Altman plots showing the difference in kcal between the reported and true intake for the MealScan3D.

Bland–Altman plots comparing of the limits of agreement between reported and true total energy intake suggest narrower limits of agreement for MS3D than for the WFR, although reporting accuracy for both the WFR and MS3D appears consistent over the range of total energy intakes (Figure 3a,b).

Table 3 compares the measurements of energy and food portion size in the MS3D and WFR arms. The validity of measuring the total energy intake (Pearson correlation) was significantly higher in the MS3D arm (*p* < 0.001); in the regression model, bias was smaller (intercept was closer to the mean) in the MS3D arm, but the regression coefficient (slope) did not differ significantly. When this result is interpreted as the percentage of true variance (r^2^) in energy explained by the two tools, MS3D explained 84.6% of true variance, a 25.3% absolute and 42.6% relative increase over the 59.3% explained by the WFR. The correlations between true and reported portion sizes for 9 of 15 individual foods (all entrees, vegetable sides, garlic bread, mashed potatoes, and cookies) were significantly larger in the MS3D arm. There were no statistically significant differences in correlations between true and reported portion sizes for either of the sauces or butters, orzo, or rice pudding. Bias was smaller (intercepts were closer to the means) for 9 of 15 foods, and the regression coefficients for 10 of 15 foods were significantly closer to 1.0 in the MS3D arm.

Table 4 compares the MS3D scanner-measured food portion sizes to participant-estimated portion sizes. These analyses are restricted to the MS3D arm only and test whether scanner-measured portion size is more accurate than participants’ estimates. The validity of measuring total energy was significantly higher (*p* < 0.001) when using scanner-measured portion size (r^2^ = 84.6% vs. 65.6%); in the regression model, bias was smaller and the slope was higher using scanner-measured portion sizes, although the difference in slopes was not statistically significant. The validity of portion size measures based on scanner measurements was significantly higher for almost all foods except the butters, apple cider glaze, and orzo. The regression intercepts were closer to the mean for almost all side dishes and desserts (garlic bread, green beans, mashed potato, cookies, orzo, carrot, rice pudding); however, for two of the three main entrees (chicken and pork), bias was significantly smaller for participant-estimated portion sizes. Slopes were significantly closer to 1.0 for 9 of 15 individual foods when portion sizes were based on the scanner measurements.

As a secondary analysis, total energy and food portions sizes in the two arms using only participants’ self-reported portion sizes and ignoring the scanner-estimated portion size in the MS3D arm were compared (Supplemental Table S2). This comparison tests whether the algorithm-driven data collection in MS3D yields data that are superior to a standard written food record. For energy, there were no differences in either validity or results from the regression models between arms. For food portion sizes, there was no consistent evidence that results differed between arms. There were few significant differences in validity or slope between the two arms, bias was comparable for the majority of foods, and the direction of differences were mixed.

## 4. Discussion

In this randomized study comparing two approaches to dietary self-report, MealScan3D more accurately captured the total energy intake and food portion sizes than a standard, written food record. The validity of measuring the total energy was significantly higher in MS3D compared to the WFR arm (0.92 vs. 0.77, respectively; *p* < 0.0001) and the mean difference between true and reported energy intake was lower in MS3D compared to the WFR arm (reported minus true kcal ± standard error: 120 ± 42 vs. −171 ± 63, respectively; *p* < 0.001).

We hypothesized two ways in which MS3D would improve the accuracy of dietary assessment: by using three-dimensional imaging to calculate portion size instead of using participant-reported portion size, and by recording food descriptions using algorithm-driven, real-time direct entry instead of unstructured written recording into a paper booklet. The findings support that three-dimensional imaging was more accurate than participant self-report, but provide no evidence that algorithm-based direct entry was superior to writing food descriptions into a paper booklet. The latter finding is consistent with a small prior study showing a comparable accuracy of energy intake recorded via a handheld personal digital assistant and traditional written food record with total energy expenditure measured by doubly labeled water [35].

Many research groups have developed image-based systems for recording food intake, though most have either used images as a memory aid for dietary recall [9,25] or have used trained dietitians or raters to estimate portion size after comparing food images to standardized images [6,7,8,10,12,27,36]. We are aware of six image-based systems that calculate food volume directly from digital images [14,15,17,23,28], though only one has reported on the accuracy of estimated energy intake. The mobile Food Record (mFR) is a smartphone app-based food record that uses standard digital images for food identification and automatic volume estimation. The ‘automated classifier’ identifies foods by comparison with reference images (of foods provided in the study), and volume is estimated by reference to a fiduciary marker, the standardization of the image angle, and fitting to pre-defined 3D geometric models [20]. An evaluation study of 45 community-dwelling adults who were provided known amounts of foods and recorded their dietary intake for 7.5 days reported a Spearman correlation of 0.58 between the mFR and doubly labeled water-assessed energy intake [28]. The mean difference between measured (mFR-estimated) and true (estimated as the difference in provided and returned food weights) energy intake was much smaller (20 kcal per day), although statistics on individual-level agreement (e.g., Pearson correlation between measured and true intake) were not reported. In addition, to our knowledge, no prior studies have tested whether individual-level agreement differs between image-based and traditional (e.g., food record) self-reported methods of food intake. Agreement among researchers on study designs and the selection of key statistics evaluating image-based dietary assessment systems are needed to move this research area forward.

The MS3D measurement of butter and sauces was poor due to both participant-related and technical issues. Participants in the MS3D arm had difficulty following the protocol for capturing images of the small plastic containers holding these foods. Participants in the MS3D arm were instructed to scan the container before and after eating each meal; however, many participants forgot to capture the images both before and after eating (*n* = 25, 27, 11, and 6 for BBQ sauce, apple cider glaze, butter (Meal 1) and butter (Meal 2), respectively). In addition, image quality was sometimes too poor to generate a three-dimensional image (*n* = 4, 2, 4, and 2 for BBQ sauce, apple cider glaze, butter (Meal 1) and butter (Meal 2), respectively) and issues with surface reflectiveness, for the sauces in particular, may have contributed to inaccurate portion size estimation.

One unexpected finding in this study was the strong correlation between true and reported intakes in the WFR group. Previous studies evaluating the validity of energy intake using weighed food intake as a criterion measure reported correlations that ranged from only 0.3 to 0.6 [37,38,39,40]. We can only speculate on why our findings were so different. Possibilities include participants’ high level of education and familiarity with research conduct, the restriction of data collection to only three meals, and the relative simplicity of the foods provided that did not include any complex food mixtures.

There are many strengths to this study. First and foremost is its use of a randomized design to support an objective comparison between MS3D and a WFR. Another strength is its avoidance of fixed household portion sizes (e.g., ½ c carrots), foods served in standard units (e.g., one apple), and packaged, standardized prepared foods, which can bias self-reported portion size. Lastly, reporting results for both the portion size of individual foods and total energy as a summary measure gives more detailed insight into the functioning of the three-dimensional imaging system. We also note some of the strengths of our technical approach. MS3D uses an infrared scanner that obviates the need for either a fiduciary marker or artificial lighting to generate three-dimensional images. Further, infrared scanning technology is now incorporated into new smartphones, and thus, the MS3D application can now work without the need for an externally mounted infrared camera. We also developed software to correct volume measurements for plate shape and depth, which does not require reference to standardized shapes when computing the volume of the three-dimensional image.

Limitations include the lack of generalizability due to the high level of participant education, limited collection of participant data which precludes the evaluation of differences related to participant characteristics, the limited number of foods and meals evaluated, the poor measurement of sauces and butter, and our decision to ignore beverages until additional software and hardware modifications could successfully measure translucent liquids. An important technical limitation is that the MS3D system was developed for research purposes, and thus requires that foods be coded by trained technicians before nutrient analysis. It would be feasible to incorporate algorithms for user self-coding; however, it is unclear whether the precision of this approach would be satisfactory for research use.

## 5. Conclusions

This study demonstrates the feasibility and accuracy of using MealScan3D, a three-dimensional image-based approach to measure dietary intake. The validity of total energy intake and food volume estimation was higher for MealScan3D than for the written food record. For most foods tested, estimates of food volumes based on three-dimensional imaging via MealScan3D were more accurate than self-report. Further technical developments, for example, the ability to capture beverages and condiments and an approach for capturing foods eaten without a plate, are needed to make the MS3D system useful in diverse eating situations.

## Figures and Tables

**Table 1:** Participant demographic characteristics and food-related responsibilities, by study arm.

	MS3D *n* = 87	WFR *n* = 92	*p*-Value	Total *n* = 179
	Mean ± SD	Mean ± SD		Mean ± SD
Age (yrs)	33.8 ± 11.0	35.1 ± 12.2	0.31	33.6 ± 11.5
	*n* (%)	*n* (%)		*n* (%)
Sex				
Male	18 (20.7)	31 (33.7)	0.05	49 (27.4)
Female	69 (79.3)	61 (66.3)		130 (72.6)
Education				
High School or Less	12 (13.8)	12 (13.0)	0.14	24 (13.4)
College	57 (65.5)	49 (53.3)		106 (59.6)
Graduate/Professional	18 (20.7)	31 (33.7)		49 (27.4)
Responsible for Food Shopping				
All or most	61 (70.1)	64 (70.0)	0.98	125 (69.8)
Some or None	26 (29.9)	24 (30.0)		54 (31.2)
Responsible for Food Preparation				
All or most	57 (66.3)	60 (65.9)	0.96	117 (66.1)
Some or None	29 (33.7)	31 (34.1)		60 (33.9)
Responsible for Meal Planning				
All or most	60 (69.8)	61 (67.0)	0.70	121 (68.4)
Some or None	26 (30.2)	30 (33.0)		56 (31.6)
Ethnicity				
Hispanic or Latino	5 (5.8)	11 (12.0)	0.15	16 (9.0)
Not Hispanic or Latino	81 (94.2)	81 (88.0)		162 (91.0)
Race				
Asian	20 (23.0)	18 (19.6)	0.34	38 (21.6)
White	53 (60.9)	61 (66.3)		114 (64.8)
Other	12 (16.1)	10 (12.1)		24 (13.6)

**Table 2:** Mean true and reported and difference between true and reported energy intake and food portion sizes, comparing MealScanner3D (MS3D) arm to written food record (WFR) arm.

	MS3D				WFR				
	*n*	True Mean ± SD	Reported Mean ± SD	Difference Mean ± SE	*n*	True Mean ± SD	Reported Mean ± SD	Difference Mean ± SE	*p*-Value for Absolute Difference between Arms
Total Energy ^a^ (kcal)	87	2671 ± 879	2791 ± 981	120 ± 42 ^c^	92	2800 ± 884	2628 ± 928	−171 ± 63 ^c^	<0.0001
Meal 1									
Lasagna (g)	87	221.9 ± 93.4	300.7 ± 139.5	78.7 ± 6.3 ^d^	92	240.7 ± 97.3	290.5 ± 155.8	49.8 ± 14.8 ^c^	0.07
Broccoli (g)	86	78.4 ± 36.4	83.5 ± 38.9	5.1 ± 1.8 ^c^	91	75.7 ± 31.7	164.0 ± 85.2	88.3 ± 7.1 ^d^	<0.0001
Garlic Bread (g)	86	77.3 ± 39.2	64.8 ± 34.9	−12.5 ± 1.8 ^d^	89	75.7 ± 34.6	55.9 ± 36.8	−19.7 ± 4.0 ^d^	0.10
Meal 2									
Chicken (g)	85	129.5 ± 63.0	174.3 ± 91.4	44.8 ± 5.4 ^d^	91	139.2 ± 62.0	154.5 ± 70.5	15.3 ± 5.9 ^b^	<0.001
BBQ Sauce (g)	53	43.9 ± 28.9	59.7 ± 34.3	15.7 ± 2.9 ^d^	80	49.4 ± 53.9	52.1 ± 61.6	2.7 ± 5.1	0.03
Green Beans (g)	83	123.9 ± 65.9	127.3 ± 71.3	3.4 ± 3.5	90	128.6 ± 68.1	165.6 ± 99.8	37.0 ± 6.2 ^d^	<0.0001
Mashed Potatoes (g)	85	172.6 ± 80.7	200.7 ± 95.9	28.1 ± 4.0 ^d^	90	177.3 ± 82.1	252.7 ± 163.1	75.4 ± 11.5 ^d^	<0.001
Butter (g)	41	6.5 ± 4.9	19.4 ± 12.2	12.9 ± 1.6 ^d^	61	8.8 ± 6.5	12.9 ± 10.8	4.1 ± 1.1 ^d^	<0.0001
Cookies (g)	76	68.4 ± 22.3	96.5 ± 40.4	28.0 ± 3.4 ^d^	86	63.8 ± 22.7	39.2 ± 39.7	−24.6 ± 4.2 ^d^	0.52
Meal 3									
Pork Loin (g)	83	127.9 ± 61.5	177.4 ± 89.9	49.5 ± 4.5 ^d^	91	134.8 ± 65.2	147.1 ± 76.0	12.3 ± 5.8 ^b^	<0.0001
Apple cider glaze (g)	51	37.4 ± 24.0	38.2 ± 25.2	0.8 ± 2.9	83	34.6 ± 26.3	37.2 ± 37.3	2.6 ± 3.2	0.69
Roasted Carrots (g)	85	138.9 ± 76.4	134.3 ± 83.3	−4.6 ± 3.1	91	138.7 ± 74.8	164.6 ± 101.3	25.9 ± 6.8 ^d^	0.005
Orzo (g)	86	126.4 ± 61.8	123.5 ± 64.3	−2.9 ± 3.7	91	135.6 ± 66.2	160.3 ± 90.7	24.7 ± 6.2 ^d^	0.003
Butter (g)	25	7.2 ± 5.1	19.4 ± 12.5	12.2 ± 2.1 ^d^	34	7.2 ± 6.2	10.1 ± 7.2	2.9 ± 1.3 ^b^	<0.001
Rice Pudding (g)	64	133.7 ± 87.1	140.2 ± 91.5	6.5 ± 4.0	83	117.4 ± 80.4	161.2 ± 160.0	43.9± 10.1 ^d^	<0.001

^a^ Total energy for all meals combined; mean difference between true and reported ^b^ *p* ≤ 0.05, ^c^ *p* ≤ 0.01, and ^d^ *p* ≤ 0.001.

**Table 3:** Associations (Pearson correlations and regression models) between true and reported total energy and food portion sizes (mean centered), comparing the MealScanner3D (MS3D) and written food record (WFR) arms ^a^.

	Validity (Pearson Correlations) ^b^			Regression Models ^c^				
				Intercept ^d^ ± SE		Slope ± SE		
	MS3D	WFR	*p*-Value for Difference	MS3D	WFR	MS3D	WFR	*p*-Value for Difference ^f^
Total Energy ^e^ (kcal)	0.92	0.77	<0.0001	2613 ± 50	2869 ± 49	0.82± 0.05	0.74 ± 0.05	0.28
Meal 1								
Lasagna (g)	0.95	0.45	<0.0001	218.6 ± 7.1	242.1 ± 6.9	0.63 ± 0.05	0.28 ± 0.04	<0.0001
Broccoli (g)	0.91	0.68	<0.0001	113.5 ± 3.1	65.8 ± 2.3	0.85 ± 0.06	0.25 ± 0.02	<0.0001
Garlic Bread (g)	0.90	0.44	<0.0001	72.7 ± 2.7	77.5 ± 2.7	1.01 ± 0.08	0.42 ± 0.07	<0.0001
Meal 2								
Chicken (g)	0.86	0.64	<0.001	123.4 ± 4.5	144.6 ± 4.3	0.59 ± 0.05	0.57 ± 0.06	0.75
BBQ Sauce (g)	0.79	0.69	0.22	40.6 ± 3.2	50.4 ± 2.6	0.66 ± 0.09	0.40 ± 0.04	0.01
Green Beans (g)	0.90	0.82	0.04	140.1 ± 4.0	118.1 ± 3.7	0.83 ± 0.05	0.56 ± 0.04	<0.0001
Mashed Potatoes (g)	0.93	0.80	<0.001	193.4 ± 4.7	167.0 ± 4.4	0.78 ± 0.05	0.40 ± 0.03	<0.0001
Butter (g)	0.58	0.64	0.66	5.6 ± 0.8	9.8 ± 0.6	0.24 ± 0.06	0.39± 0.06	0.07
Cookies (g)	0.71	0.30	<0.001	56.6 ± 2.8	68.4 ± 2.5	0.39 ± 0.06	0.17 ± 0.05	<0.01
Meal 3								
Pork Loin (g)	0.92	0.70	<0.0001	117.9 ± 4.2	143.4 ± 4.1	0.63 ± 0.05	0.60 ± 0.05	0.68
Apple cider glaze (g)	0.65	0.64	0.92	37.7 ± 2.7	35.3 ± 2.2	0.62 ± 0.13	0.45 ± 0.06	0.18
Roasted Carrots (g)	0.94	0.76	<0.0001	152.4 ± 4.3	130.5 ± 4.2	0.86 ± 0.05	0.56 ± 0.04	<0.0001
Orzo (g)	0.85	0.76	0.08	141.8 ± 4.3	125.7 ± 4.1	0.82 ± 0.06	0.55 ± 0.04	0.001
Butter (g)	0.54	0.51	0.87	6.0 ± 1.1	8.7 ± 1.0	0.22 ± 0.08	0.38 ± 0.11	0.25
Rice Pudding (g)	0.94	0.92	0.41	144.3 ± 4.0	113.1 ± 3.5	0.89 ± 0.04	0.46 ± 0.02	<0.0001

^a^ Sample sizes in each cell are given in Table 2. ^b^ All correlations *p* < 0.01. ^c^ Intercepts and regression coefficients from model: True = Arm (coded 0 or 1) + mean centered Reported Value + (Arm × mean centered Reported Value). ^d^ Total energy and individual food portions sizes centered on the overall mean values as follows: total energy: 2721 kcal; lasagna: 295.5 g; broccoli: 124.9 g; garlic bread: 60.3 g; chicken: 164.1; BBQ sauce: 54.7 g; green beans: 146.8 g; potatoes: 227.3 g; butter (Meal 2): 15.5; cookies: 66.1 g; pork: 161.5 g; apple cider glaze: 38.7 g; carrots: 150 g; orzo: 142.4 g; butter (Meal 3): 14.1 g; pudding: 152.1 g. ^e^ Total energy for all meals combined. ^f^ *p*-value testing for differences in slopes between the MS3D and WFR arms.

**Table 4:** Associations (Pearson correlations and regression models) between true and reported total energy and food portion sizes (mean centered), comparing 3D scanner-measurements to participant-estimated portion sizes in the MealScanner3D (MS3D) arm only ^a^.

	Pearson Correlations ^b^			Regression Models ^c^				
				Intercept ^d^ ± SE		Slope ± SE		
	Scanner- Measured	Participant- Estimated	*p*-Value for Difference	Scanner-Measured	Participant- Estimated	Scanner-Measured	Participant- Estimated	*p*-Value for Difference ^f^
Total Energy ^e^ (kcal)	0.92	0.81	<0.001	2619 ± 38	2821 ± 57	0.72 ± 0.04	0.63 ± 0.05	0.06
Meal 1								
Lasagna (g)	0.95	0.71	<0.001	218.1 ± 3.2	224.5 ± 7.1	0.60 ± 0.02	0.41 ± 0.05	<0.001
Broccoli (g)	0.91	0.73	<0.001	80.7 ± 1.7	76.6 ± 2.7	0.74 ± 0.04	0.58 ± 0.07	0.02
Garlic Bread (g)	0.90	0.74	<0.001	73.7 ± 1.8	80.4 ± 2.9	0.87 ± 0.05	0.75 ± 0.09	0.15
Meal 2								
Chicken (g)	0.86	0.73	<0.01	121.9 ± 3.6	134.5 ± 4.8	0.53 ± 0.04	0.47 ± 0.06	0.36
BBQ Sauce (g)	0.79	0.42	<0.001	49.7 ± 2.6	45.8 ± 3.4	0.47 ± 0.06	0.18 ± 0.06	<0.001
Green Beans (g)	0.90	0.79	0.001	138.4 ± 3.3	113.8 ± 4.6	0.73 ± 0.04	0.44 ± 0.04	<0.001
Mashed Potatoes (g)	0.93	0.81	<0.001	185.3 ± 3.3	164.1 ± 5.3	0.71 ± 0.04	0.44 ± 0.04	<0.001
Butter (g)	0.58	0.71	0.26	6.0 ± 0.7	6.7 ± 0.6	0.11 ± 0.04	0.11 ± 0.04	0.83
Cookies (g)	0.71	0.42	<0.001	59.8 ± 2.1	78.7 ± 5.2	0.24 ± 0.03	0.29 ± 0.12	0.59
Meal 3								
Pork Loin (g)	0.92	0.77	<0.001	119.5 ± 2.7	136.4 ± 4.3	0.59 ± 0.03	0.58 ± 0.05	0.79
Apple cider glaze (g)	0.65	0.39	0.06	41.1 ± 2.5	37.2 ± 3.2	0.35 ± 0.07	0.07 ± 0.06	<0.001
Roasted Carrots (g)	0.94	0.85	<0.001	154.4 ± 2.9	123.9 ± 4.6	0.81 ± 0.03	0.63 ± 0.05	<0.001
Orzo (g)	0.85	0.79	0.09	138.4 ± 3.6	117.1 ± 4.3	0.64 ± 0.05	0.43 ± 0.04	<0.001
Butter (g)	0.54	0.77	0.11	6.9 ± 0.9	8.0 ± 0.8	0.10 ± 0.05	0.19 ± 0.06	0.12
Rice Pudding	0.94	0.74	<0.001	146.3 ± 3.9	122.1 ± 7.7	0.78 ± 0.04	0.46 ± 0.06	<0.001

^a^ Sample sizes in each cell are given in Table 2. ^b^ All correlations *p* < 0.01. ^c^ Model: True = measurement type (scanner measure vs. participant estimate, coded 0 or 1) + mean centered measured value + (measurement type × mean centered measured value); intercept and slope estimates from seemingly unrelated regression models. ^d^ Total energy and individual food portions sizes centered on the overall mean value as follows: total energy: 2637 kcal; lasagna: 294.2 g; broccoli: 86.6 g; garlic bread: 60.7 g; chicken: 160.0; BBQ sauce: 71.9 g; green beans: 147.3 g; potatoes: 218.7 g; butter (Meal 2): 16.5; cookies: 64.3 g; pork: 163.3 g; apple cider glaze: 46.6 g; carrots: 153.4 g; orzo: 142.2 g; butter (Meal 3): 16.5 g; pudding: 156.3 g. ^e^ Total energy for all meals combined. ^f^ *p*-value testing for differences in slopes between the MS3D and WFR arms.

## Data Availability

Data are contained within the article and Supplementary Materials.

## Supplementary Materials

The following supporting information can be downloaded at https://www.mdpi.com/article/10.3390/nu16060828/s1: Table S1: Descriptions of study foods; Table S2: Associations (Pearson correlations and regression models) between true and participant-estimated total energy and food portion sizes (mean centered), comparing MealScanner3D (MS3D) to the written food record (WFR) arm.

## Appendix A

**MealScan3D food volume estimation methods**
**3D Scanning**

The Structure Sensor uses a structured-light system to capture 3D scans of small objects. The sensor emits a calibrated infrared light pattern and records the pattern as it deforms and scatters off nearby objects. The reflected pattern is encoded as a two-dimensional (2D) depth image where the intensity of each pixel in the image maps to the distance from the object to the sensor. A color image is obtained simultaneously, such that each pixel in the color image maps to its corresponding pixel in the depth image. Multiple consecutive depth and color images are fused together by the sensor to obtain a 3D scan.

Users are instructed to capture a 5 s scan of their meal from a top-down view, such that the table surface always appears furthest away from the sensor. Any objects on the surface of the table always appear closer to the sensor than the table surface. Scans are represented using a 3D coordinate system such that the table surface is roughly co-planar with the z=0 plane and any objects on the surface of the table are represented by a strictly positive function f(x,y).

**Segmentation**

A pre-processing algorithm attempts to automatically identify any plate/container boundaries (plates, bowls, etc.) by searching for ellipses in each scan. The boundary of each detected plate/container is superimposed on the scan, and the nutritional coder rejects any false positives or manually corrects any misaligned true positives. Verified plate/container boundaries are stored and used as templates for container searches in subsequent scans.

The food boundaries within plates/containers are estimated by searching for distinct color and depth changes within the plate/container boundary. More prone to error than plate/container detection, food boundaries can be corrected or entirely re-traced by a nutritional coder. Once the food boundary is known, each point within a plate/container is classified as either a point on a food or a point on the plate/container.

**Reconstruction**

Plates/containers with radial symmetry are described in 3D using the height of the plate/container above the table as a function of radial distance from the center of the plate/container. If a continuous path can be traced from the center of a plate/container to its edge without intersecting a food item, the entire surface of the plate/container can be reconstructed automatically. For scans with food occluding all plate/container points at a given radial distance, the coder estimates the missing height using an interactive plot of the plate/container heights at various radial distances. Unknown heights are accurately estimated using the neighboring known heights. The 3D surface of the plate/container is reconstructed using the completed 2D profile and applying radial symmetry.

In general, the boundary of a food item in contact with the plate/container surface is equivalent to the boundary of the plate/container surface in contact with the food item. However, using a top-down view for scanning, spherical foods such as apples, oranges, etc., only expose the top hemisphere of their surface to the sensor. In these cases, the boundary of an imaginary surface through the equator of the food and parallel to the table of the surface is used, and the resulting estimated volume is doubled, to account for the portion of the food not visible to the sensor.

**Volume Estimation**

The volume of a food item is estimated by considering the volume between the food surface exposed to the sensor and the reconstructed plate/container surface bounded by the exposed surface of the food. The food surface function f(x,y) is positive for all points x,y on the food surface and 0 elsewhere. Similarly, the plate/container surface function bounded by the food surface, $c_{f}\left(x,y\right)$, is positive for all non-zero values of f(x,y), and 0 elsewhere. Each volume is computed separately as the double integral over the respective surface region and the difference is taken:

$$V_{f,i}=\iint_{Sf}f\left(x,y\right)\;dx\;dy-\iint_{Sc}c_{f}\left(x,y\right)\;dx\;dy\;\;$$

A continuous 3D surface is fitted over the food surface function using cubic interpolation. The double integrals are numerically evaluated using the quad2d function in MATLAB. The total volume of each food consumed is determined by the following equation:

$$V_{f,consumed}=V_{f,start\;}+\sum V_{f,seconds}-\sum V_{f,leftovers}$$

## References

[1] Subar A.F., Kipnis V., Troiano R.P., Midthune D., Schoeller D.A., Bingham S., Sharbaugh C.O., Trabulsi J., Runswick S., Ballard-Barbash R. (2003). Using Intake Biomarkers to Evaluate the Extent of Dietary Misreporting in a Large Sample of Adults: The Open Study. Am. J. Epidemiol..

[2] Freedman L.S., Commins J.M., Moler J.E., Arab L., Baer D.J., Kipnis V., Midthune D., Moshfegh A.J., Neuhouser M.L., Prentice R.L. (2014). Pooled Results from 5 Validation Studies of Dietary Self-Report Instruments Using Recovery Biomarkers for Energy and Protein Intake. Am. J. Epidemiol..

[3] Freedman L.S., Commins J.M., Moler J.E., Willett W., Tinker L.F., Subar A.F., Spiegelman D., Rhodes D., Potischman N., Neuhouser M.L. (2015). Pooled Results from 5 Validation Studies of Dietary Self-Report Instruments Using Recovery Biomarkers for Potassium and Sodium Intake. Am. J. Epidemiol..

[4] Prentice R.L., Tinker L.F., Huang Y., Neuhouser M.L. (2013). Calibration of Self-Reported Dietary Measures Using Biomarkers: An Approach to Enhancing Nutritional Epidemiology Reliability. Curr. Atheroscler. Rep..

[5] Williamson D.A., Allen H.R., Martin P.D., Alfonso A., Gerald B., Hunt A. (2004). Digital Photography: A New Method for Estimating Food Intake in Cafeteria Settings. Eat. Weight. Disord..

[6] Pettitt C., Liu J., Kwasnicki R.M., Yang G.-Z., Preston T., Frost G. (2016). A Pilot Study to Determine Whether Using a Lightweight, Wearable Micro-Camera Improves Dietary Assessment Accuracy and Offers Information on Macronutrients and Eating Rate. Br. J. Nutr..

[7] Martin C.K., Han H., Coulon S.M., Allen H.R., Champagne C.M., Anton S.D. (2009). A Novel Method to Remotely Measure Food Intake of Free-Living Individuals in Real Time: The Remote Food Photography Method. Br. J. Nutr..

[8] Casperson S.L., Sieling J., Moon J., Johnson L., Roemmich J.N., Whigham L. (2015). A Mobile Phone Food Record App to Digitally Capture Dietary Intake for Adolescents in a Free-Living Environment: Usability Study. JMIR mHealth uHealth.

[9] Ptomey L.T., Willis E.A., Honas J.J., Mayo M.S., Washburn R.A., Herrmann S.D., Sullivan D.K., Donnelly J.E. (2015). Validity of Energy Intake Estimated by Digital Photography Plus Recall in Overweight and Obese Young Adults. J. Acad. Nutr. Diet..

[10] E Rollo M., Ash S., Lyons-Wall P., Russell A. (2011). Trial of a Mobile Phone Method for Recording Dietary Intake in Adults with Type 2 Diabetes: Evaluation and Implications for Future Applications. J. Telemed. Telecare.

[11] Wang D.-H., Kogashiwa M., Kira S. (2006). Development of a New Instrument for Evaluating Individuals’ Dietary Intakes. J. Am. Diet. Assoc..

[12] Gemming L., Doherty A., Kelly P., Utter J., Ni Mhurchu C. (2013). Feasibility of a Sensecam-Assisted 24-h Recall to Reduce under-Reporting of Energy Intake. Eur. J. Clin. Nutr..

[13] Lu Y., Stathopoulou T., Vasiloglou M.F., Pinault L.F., Kiley C., Spanakis E.K., Mougiakakou S. (2020). goFOOD^TM^: An Artificial Intelligence System for Dietary Assessment. Sensors.

[14] Rhyner D., Loher H., Dehais J., Anthimopoulos M., Shevchik S., Botwey R.H., Duke D., Stettler C., Diem P., Mougiakakou S. (2016). Carbohydrate Estimation by a Mobile Phone-Based System Versus Self-Estimations of Individuals with Type 1 Diabetes Mellitus: A Comparative Study. J. Med. Internet Res..

[15] Kong F., Tan J. (2012). Dietcam: Automatic Dietary Assessment with Mobile Camera Phones. Pervasive Mob. Comput..

[16] Puri M., Zhu Z., Yu Q., Divakaran A., Sawhney H. Recognition and Volume Estimation of Food Intake Using a Mobile Device. Proceedings of the 2009 Workshop on Applications of Computer Vision (WACV).

[17] Zhang W., Yu Q., Siddiquie B., Divakaran A., Sawhney H. (2015). “Snap-N-Eat”: Food Recognition and Nutrition Estimation on a Smartphone. J. Diabetes Sci. Technol..

[18] Fang S., Zhu F., Jiang C., Zhang S., Boushey C.J., Delp E.J. A Comparison of Food Portion Size Estimation Using Geometric Models and Depth Images. Proceedings of the 2016 IEEE International Conference on Image Processing (ICIP).

[19] Makhsous S., Mohammad H.M., Schenk J.M., Mamishev A.V., Kristal A.R. (2019). A Novel Mobile Structured Light System in Food 3d Reconstruction and Volume Estimation. Sensors.

[20] Fang S., Liu C., Zhu F., Delp E.J., Boushey C.J. (2015). Single-View Food Portion Estimation Based on Geometric Models. ISM.

[21] Chae J., Woo I., Kim S., Maciejewski R., Zhu F., Delp E.J., Boushey C.J., Ebert D.S. (2011). Volume Estimation Using Food Specific Shape Templates in Mobile Image-Based Dietary Assessment. Proc. SPIE Int. Soc. Opt. Eng..

[22] Zhu F., Bosch M., Woo I., Kim S., Boushey C.J., Ebert D.S., Delp E.J. (2010). The Use of Mobile Devices in Aiding Dietary Assessment and Evaluation. IEEE J. Sel. Top. Signal Process..

[23] Jia W., Chen H.-C., Yue Y., Li Z., Fernstrom J., Bai Y., Li C., Sun M. (2014). Accuracy of Food Portion Size Estimation from Digital Pictures Acquired by a Chest-Worn Camera. Public Health Nutr..

[24] Williamson D.A., Allen H.R., Martin P.D., Alfonso A.J., Gerald B., Hunt A. (2003). Comparison of Digital Photography to Weighed and Visual Estimation of Portion Sizes. J. Am. Diet. Assoc..

[25] Gemming L., Rush E., Maddison R., Doherty A., Gant N., Utter J., Ni Mhurchu C. (2015). Wearable Cameras Can Reduce Dietary under-Reporting: Doubly Labelled Water Validation of a Camera-Assisted 24 h Recall. Br. J. Nutr..

[26] Martin C.K., Correa J.B., Han H., Allen H.R., Rood J.C., Champagne C.M., Gunturk B.K., Bray G.A. (2012). Validity of the Remote Food Photography Method (RFPM) for Estimating Energy and Nutrient Intake in near Real-Time. Obesity.

[27] Rollo M.E., Ash S., Lyons-Wall P., Russell A.W. (2015). Evaluation of a Mobile Phone Image-Based Dietary Assessment Method in Adults with Type 2 Diabetes. Nutrients.

[28] Boushey C.J., Spoden M., Delp E.J., Zhu F., Bosch M., Ahmad Z., Shvetsov Y.B., DeLany J.P., Kerr D.A. (2017). Reported Energy Intake Accuracy Compared to Doubly Labeled Water and Usability of the Mobile Food Record among Community Dwelling Adults. Nutrients.

[29] Kawano Y., Yanai K. Real-Time Mobile Food Recognition System. Proceedings of the IEEE Conference on Computer Vision and Pattern Recognition Workshops.

[30] He H., Kong F., Tan J. (2016). Dietcam: Multiview Food Recognition Using a Multikernel Svm. IEEE J. Biomed. Health Inform..

[31] Kolar A.S., Patterson R.E., White E., Neuhouser M.L., Frank L.L., Standley J., Potter J.D., Kristal A.R. (2005). A Practical Method for Collecting 3-Day Food Records in a Large Cohort. Epidemiology.

[32] Bland J.M., Altman D.G. (1986). Statistical Methods for Assessing Agreement between Two Methods of Clinical Measurement. Lancet.

[33] Weaver B., Wuensch K.L. (2013). SPSS and SAS Programs for Comparing Pearson Correlations and OLS Regression Coefficients. Behav. Res. Methods.

[34] SAS Institute Inc. (2014). Sas/Ets® 13.2 User’s Guide. The Syslin Procedure.

[35] McClung H.L., Sigrist L.D., Smith T.J., Karl J.P., Rood J.C., Young A.J., Bathalon G.P. (2009). Monitoring Energy Intake: A Hand-Held Personal Digital Assistant Provides Accuracy Comparable to Written Records. J. Am. Diet. Assoc..

[36] Nyström C.D., Forsum E., Henriksson H., Trolle-Lagerros Y., Larsson C., Maddison R., Timpka T., Löf M. (2016). A Mobile Phone Based Method to Assess Energy and Food Intake in Young Children: A Validation Study against the Doubly Labelled Water Method and 24 h Dietary Recalls. Nutrients.

[37] Yuan C., Spiegelman D., Rimm E.B., Rosner B.A., Stampfer M.J., Barnett J.B., Chavarro J.E., Subar A.F., Sampson L.K., Willett W.C. (2017). Validity of a Dietary Questionnaire Assessed by Comparison with Multiple Weighed Dietary Records or 24-h Recalls. Am. J. Epidemiol..

[38] Rosilene W., Cumming R., Travison T., Blyth F., Naganathan V., Allman-Farinelli M., Hirani V. (2015). Relative Validity of a Diet History Questionnaire against a Four-Day Weighed Food Record among Older Men in Australia: The Concord Health and Ageing in Men Project (Champ). J. Nutr. Health Aging.

[39] Willett W.C., Sampson L., Stampfer M.J., Rosner B., Bain C., Witschi J., Hennekens C.H., Speizer F.E. (1985). Reproducibility and Validity of a Semiquantitative Food Frequency Questionnaire. Am. J. Epidemiol..

[40] Al-Shaar L., Yuan C., Rosner B., Dean S.B., Ivey K.L., Clowry C.M., A Sampson L., Barnett J.B., Rood J., Harnack L.J. (2021). Reproducibility and Validity of a Semiquantitative Food Frequency Questionnaire in Men Assessed by Multiple Methods. Am. J. Epidemiol..

