# Supplementary material for: The Use of Three-Dimensional Images and Food Descriptions from a Smartphone Device Is Feasible and Accurate for Dietary Assessment
Source: Nutrients. 2024 Mar 14;16(6):828. doi: 10.3390/nu16060828 (PMC10976213; doi:10.3390/nu16060828)
Supplement: Supplementary file 1 [file nutrients-16-00828-s001.zip › nutrients-2886616-supplementary.pdf]

Supplementary Materials

Table S1. Descriptions of Study foods.

	**Amount of food provided to participant**		**Energy density of food**
	**Mean**	**(min - max)**	**Energy/100 g**
**Meal 1**			
Lasagna	849.0 g	(684 – 1025)	202 kcal
Broccoli	201.0 g	(140 – 273)	87 kcal
Garlic Bread	183.1 g	(138 – 258)	337 kcal
**Meal 2**			
Chicken	223.5 g	(155 – 332)	163 kcal
BBQ Sauce	163.2 g	(96 – 267)	81 kcal
Green Beans	251.8 g	(161 – 371)	59 kcal
Mashed Potatoes	342.7 g	(246 – 472)	141 kcal
Butter	22.8 g	(12 – 48)	717 kcal
Cookies	79.3 g	(46 – 124)	470 kcal
**Meal 3**			
Pork Loin	236.2 g	(156 – 357)	207 kcal
Apple cider glaze	136.8 g	(74 – 249)	106 kcal
Roasted Carrots	275.7 g	(160 – 332)	144 kcal
Orzo	244.0 g	(160 – 400)	256 kcal
Butter	23.4 g	(13 – 41)	717 kcal
Rice Pudding	246.4 g	(154 – 389)	196 kcal

Table S2. Associations (Pearson correlations and regression models) between true and participant-estimated total energy and food portion sizes (mean centered), comparing MealScanner 3-D (MS3D) to Written Food Record (WFR) arms [a]

	Pearson Correlations[b]			Regression Models[c]					
				Intercept[d] ± se			Slope ± se		
	MS3D	WFR	p-value for difference	MS3D	WFR		MS3D	WFR	p-value for difference[f]
Total Energy[e] (kcal)	0.81	0.77	0.46	2843 ± 60	2869 ± 57		0.72 ± 0.06	0.74 ± 0.06	0.81
**Meal 1**									
Lasagna (g)	0.71	0.45	0.01	222.3 ± 8.4	242.1 ± 8.1		0.44 ± 0.06	0.28 ± 0.05	0.04
Broccoli (g)	0.73	0.68	0.50	102.4 ± 3.5	65.8 ± 2.8		0.68 ± 0.07	0.25 ± 0.03	<0.0001
Garlic Bread (g)	0.74	0.44	0.002	80.6 ± 3.2	77.5 ± 3.1		0.90 ± 0.10	0.42 ± 0.08	0.0003
**Meal 2**									
Chicken (g)	0.73	0.64	0.27	137.3 ± 5.0	144.6 ± 4.8		0.55 ± 0.06	0.57 ± 0.07	0.88
BBQ Sauce (g)	0.42	0.69	0.01	40.1 ± 3.0	50.4 ± 3.0		0.14 ± 0.03	0.40 ± 0.05	<0.0001
Green Beans (g)	0.79	0.82	0.66	111.8 ± 4.4	118.1 ± 4.3		0.51 ± 0.04	0.56 ± 0.04	0.43
Mashed Potatoes (g)	0.81	0.80	0.94	174.7 ± 5.2	167.0 ± 5.2		0.49 ± 0.04	0.40 ± 0.03	0.10
Butter (g)	0.71	0.64	0.49	7.6 ± 0.6	9.8 ± 0.6		0.31 ± 0.05	0.39 ± 0.05	0.27
Cookies (g)	0.42	0.30	0.42	88.9 ± 6.3	68.4 ± 2.8		0.52 ± 0.15	0.17 ± 0.06	0.03
**Meal 3**									
Pork Loin (g)	0.77	0.70	0.29	137.4 ± 4.9	143.4 ± 4.7		0.63 ± 0.06	0.60 ± 0.06	0.73
Apple cider glaze (g)	0.39	0.64	0.03	35.9 ± 2.7	35.3 ± 2.5		0.24 ± 0.06	0.45 ± 0.07	0.016
Roasted Carrots (g)	0.85	0.76	0.11	120.1 ± 5.0	141.5 ± 4.7		0.67 ± 0.05	0.56 ± 0.05	0.11
Orzo (g)	0.79	0.76	0.59	114.0 ± 4.5	125.7 ± 4.4		0.56 ± 0.05	0.55 ± 0.05	0.95
Butter (g)	0.77	0.51	0.06	8.5 ± 0.8	8.7 ± 0.9		0.46 ± 0.07	0.38 ± 0.10	0.55
Rice Pudding (g)	0.74	0.92	0.0002	117.8 ± 5.8	113.1 ± 5.1		0.53 ± 0.05	0.46 ± 0.03	0.21

[a] Sample sizes in each cell are given in Table 2. [b]All correlations $p<0.01$. [c] Intercepts and regression coefficients from model: True = Arm (coded 0 or 1) + mean centered Participant-Reported Value + (Arm * mean centered Participant-Reported Value). [d] Total energy and individual food portions sizes centered on the general mean value as follows: Total Energy: 2721 kcal; Lasagna: 295.5 g; Broccoli: 124.9 g; Garlic Bread: 60.3 g; Chicken: 164.1; BBQ sauce: 54.7g; Green Beans: 146.8g; Potatoes: 227.3g; Butter (Meal 2): 15.5; Cookies: 66.1g; Pork: 161.5g; Apple cider glaze: 38.7g; Carrots: 150g; Orzo: 142.4g; Butter (Meal 3): 14.1g; Pudding: 152.1g. [e] Total energy for all meals combined. [f] p-value testing for differences in slopes between the MS3D and WFR arms.
